# Supplementary figures and images for: Decomposing past and future: Integrated information decomposition based on shared probability mass exclusions
Source: PLoS One. 2023 Mar 23;18(3):e0282950. doi: 10.1371/journal.pone.0282950 (PMC10035902; doi:10.1371/journal.pone.0282950)

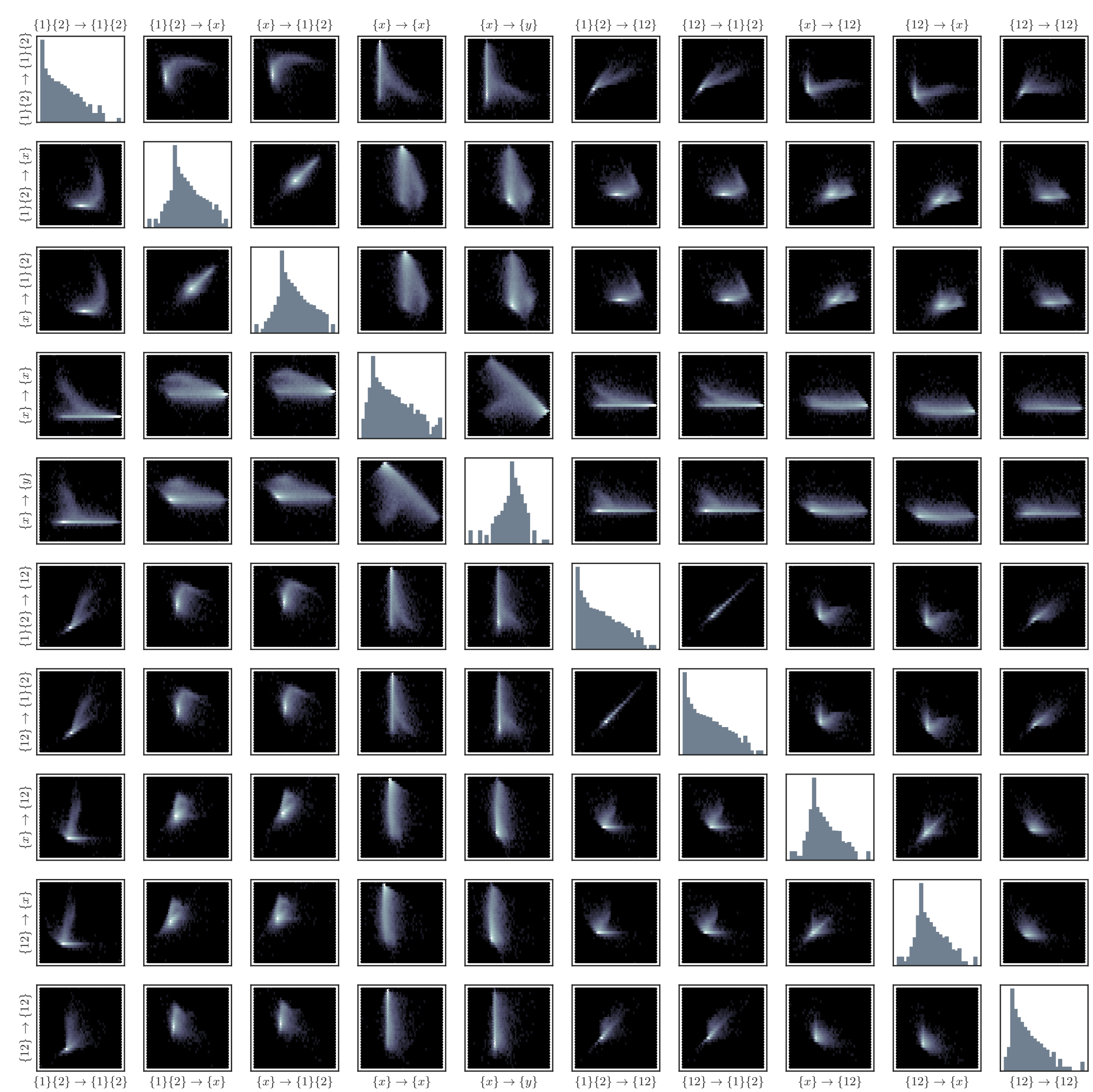

Supplement: S1 Fig — Represented as two-dimensional log-probability density hexagonal histograms. The middle diagonal replicates the histograms seen in Fig 5. The correlations between various atoms are complex and not always trivial, or linear. (PDF) [file pone.0282950.s001.pdf]
