# Supplementary material for: Decomposing past and future: Integrated information decomposition based on shared probability mass exclusions
Source: PLoS One. 2023 Mar 23;18(3):e0282950. doi: 10.1371/journal.pone.0282950 (PMC10035902; doi:10.1371/journal.pone.0282950)
Supplement: S2 Fig — Visual comparion with Fig 8 shows that the overall pattern can still be discerned despite the very noisy avalanches. (PDF) [file pone.0282950.s002.pdf]

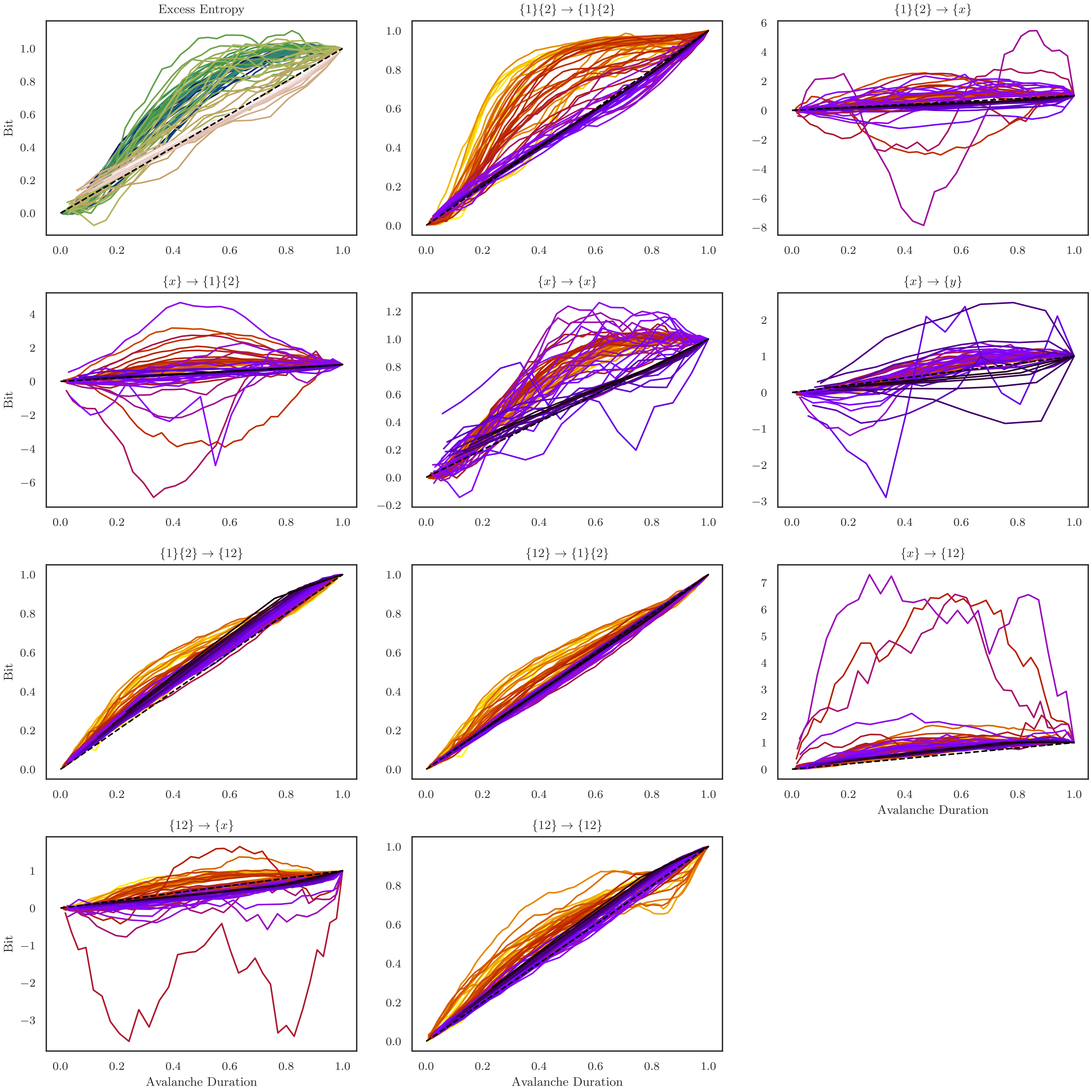
Excess Entropy
$\{1\}\{2\} \to \{1\}\{2\}$
$\{1\}\{2\} \to \{x\}$
$\{x\} \to \{1\}\{2\}$
$\{x\} \to \{x\}$
$\{x\} \to \{y\}$
$\{1\}\{2\} \to \{12\}$
$\{12\} \to \{1\}\{2\}$
$\{x\} \to \{12\}$
$\{12\} \to \{x\}$
$\{12\} \to \{12\}$
Bit
Avalanche Duration
