# Supplementary material for: Decomposing past and future: Integrated information decomposition based on shared probability mass exclusions
Source: PLoS One. 2023 Mar 23;18(3):e0282950. doi: 10.1371/journal.pone.0282950 (PMC10035902; doi:10.1371/journal.pone.0282950)
Supplement: S1 Appendix — Various proofs and a worked example of computing Iτsx. (PDF) [file pone.0282950.s003.pdf]

S1 Appendix

Appendix A: Proof of identity between formulations of $\mathbf{I_{\tau sx}}$

The proof of identity between the informative/misinformative decomposition of $I_{\tau sx}$ and the partial entropy decomposition is straightforward. I begin with the definition of the informative and misinformative components of $i_{\tau sx}$, and the relationship between them:

$$\begin{aligned}
i^{+}_{\tau sx}(\boldsymbol{\alpha} \rightarrow \boldsymbol{\beta}) &= \log_2 \frac{1}{P(\boldsymbol{\alpha})} \\
i^{-}_{\tau sx}(\boldsymbol{\alpha} \rightarrow \boldsymbol{\beta}) &= \log_2 \frac{P(\boldsymbol{\beta})}{P(\boldsymbol{\alpha} \cap \boldsymbol{\beta})} \\
i_{\tau sx}(\boldsymbol{\alpha} \rightarrow \boldsymbol{\beta}) &= i^{+}_{\tau sx}(\boldsymbol{\alpha} \rightarrow \boldsymbol{\beta}) - i^{-}_{\tau sx}(\boldsymbol{\alpha} \rightarrow \boldsymbol{\beta})
\end{aligned}$$

We also have the definitions of each of the partial entropy functions as well.

$$\begin{aligned}
h_{sx}(\boldsymbol{\alpha}) &= \log_2 \frac{1}{P(\boldsymbol{\alpha})} \\
h_{sx}(\boldsymbol{\beta}) &= \log_2 \frac{1}{P(\boldsymbol{\beta})} \\
h_{sx}(\boldsymbol{\alpha} \cap \boldsymbol{\beta}) &= \log_2 \frac{1}{P(\boldsymbol{\alpha} \cap \boldsymbol{\beta})}
\end{aligned}$$

The equivalence between the informative component of $i_{\tau sx}$ and the partial entropy of the past is trivial, following from the definitions of both.

$$i^{+}_{\tau sx}(\boldsymbol{\alpha} \rightarrow \boldsymbol{\beta}) = h_{sx}(\boldsymbol{\alpha})$$

The misinformative component is less straightforward, but still simple. The misinformative information in $i_{\tau sx}$ is the difference between the probability mass exclusions induced by $\boldsymbol{\alpha} \cap \boldsymbol{\beta}$ and the exclusion induced by just $\boldsymbol{\beta}$.

This argument is equivalent to the identity that $h(x|y) = h(x, y) - h(y)$.

$$\begin{aligned}
i^{-}_{\tau sx}(\boldsymbol{\alpha} \rightarrow \boldsymbol{\beta}) &= \log_2 \frac{P(\boldsymbol{\beta})}{P(\boldsymbol{\alpha} \cap \boldsymbol{\beta})} \\
&= \log_2 P(\boldsymbol{\beta}) - \log_2 P(\boldsymbol{\alpha} \cap \boldsymbol{\beta}) \\
&= h_{sx}(\boldsymbol{\alpha} \cap \boldsymbol{\beta}) - h_{sx}(\boldsymbol{\beta})
\end{aligned}$$

Finally, basic algebra shows us that the two formulations of $i_{\tau sx}$ are equivalent.

$$\begin{aligned}
i_{\tau sx}(\boldsymbol{\alpha} \rightarrow \boldsymbol{\beta}) &= i^{+}_{\tau sx}(\boldsymbol{\alpha} \rightarrow \boldsymbol{\beta}) - i^{-}_{\tau sx}(\boldsymbol{\alpha} \rightarrow \boldsymbol{\beta}) \\
&= h_{sx}(\boldsymbol{\alpha}) - \Big[- h_{sx}(\boldsymbol{\beta}) + h_{sx}(\boldsymbol{\alpha} \cap \boldsymbol{\beta}) \Big] \\
&= h_{sx}(\boldsymbol{\alpha}) + h_{sx}(\boldsymbol{\beta}) - h_{sx}(\boldsymbol{\alpha} \cap \boldsymbol{\beta}) \square
\end{aligned}$$

Appendix B: Proof that the partial entropy decomposition has no misinformative component

The decomposition of $h(\mathbf{x})$, begins with the identity that $i(\mathbf{x}; \mathbf{x}) = h(\mathbf{x})$. Using $i_{sx}(x^1, \ldots, x^k; \mathbf{x})$, the partial information can be decomposed into informative and

misinformative components. In this case, I will show that $i_{sx}(x^1, \ldots, x^k; \mathbf{x}) = i^+_{sx}(x^1, \ldots, x^k; \mathbf{x})$: there is only an informative component.

Given:

$$i^+_{sx}(x^1, \ldots, x^k; \mathbf{x}) = \log_2 \frac{1}{P(x^1 \cup \ldots \cup x^k)} \\ = h_{sx}(x^1, \ldots, x^k) \tag{26}$$

It suffices to show that $i^-_{sx}(x^1, \ldots, x^k; \mathbf{x}) = 0$ in all cases.

From the definition (Eq. 17):

$$i^-_{sx}(x^1, \ldots, x^k; \mathbf{x}) = \log_2 \frac{P(\mathbf{x})}{P(\mathbf{x} \cap (x^1 \cup \ldots \cup x^k))} \tag{27}$$

Since $\mathbf{x} = \{x^1 \cap \ldots \cap x^k\}$:

$$i^-_{sx}(x^1, \ldots, x^k; \mathbf{x}) = \\ \log_2 \frac{P(x^1 \cap \ldots \cap x^k)}{P((x^1 \cap \ldots \cap x^k) \cap (x^1 \cup \ldots \cup x^k))} \tag{28}$$

and $(x^1 \cap \ldots \cap x^n) \cap (x^1 \cup \ldots \cup x^k) = (x^1 \cap \ldots \cap x^k)$, so Eq. 28 reduces to:

$$i^-_{sx}(x^1, \ldots, x^k) = \log_2 \frac{P(x^1 \cap \ldots \cap x^k)}{P(x^1 \cap \ldots \cap x^k)} \tag{29}$$

Which equals 0 bit. □

A similar proof is provided in [41].

Appendix C: $\mathbf{H_{sx}(\alpha \cap \beta)}$ Worked Example

P	$(X^1$	$X^2)$	$\rightarrow$	$(Y^1$	$Y^2)$
1/16	0	0		0	0
1/16	0	0		0	1
1/16	0	0		1	0
1/16	0	0		1	1
1/16	0	1		0	0
1/16	0	1		0	1
1/16	0	1		1	0
1/16	0	1		1	1
1/16	1	0		0	0
1/16	1	0		0	1
1/16	1	0		1	0
1/16	1	0		1	1
1/16	1	1		0	0
1/16	1	1		0	1
1/16	1	1		1	0
1/16	1	1		1	1

Table 2. Example state-transition probability table.

Here I will provide a simple, worked example of the $i_{\tau\, sx}$ calculation to demonstrate some of it's essential features. Consider the transition matrix displayed in Table 2: I

will begin with the double-redundancy: $i_{\tau sx}(\{1\}\{2\} \rightarrow \{1\}\{2\})$. Suppose that I learn that $X^1 = 0 \vee X^2 = 0$ and $Y^1 = 0 \vee Y^2 = 0$.

We begin with the marginal entropy decomposition on $\mathbf{x}$. Learning $X^1 = 0 \vee X^2 = 0$ excludes all transitions consistent with $X^1 = 1 \wedge X^2 = 1$: $(1,1) \rightarrow (0,0)$, $(1,1) \rightarrow (0,1)$, etc, and leaves twelve possibilities remaining. The resulting partial entropy value is $-\log_2(3/4)$ bit, and likewise for $Y^1 = 0 \vee Y^2 = 0$. This highlights the first unusual aspect of the partial entropy decomposition: independent variables can share entropy. While initially counter-intuitive, this makes sense when considering that $h_{sx}(x^1, x^2)$ does not necessarily quantify a statistical dependence *between* x^1 and x^2 the way mutual information does, but rather the dependence between x^1, x^2 and the joint state of x^1 and x^2 together (this is equivalent to the notorious two-bit copy gate).

The intersection entropy $h_{sx}((x^1 \cup x^2) \cap (y^1 \cup y^2))$ is computed by excluding all transitions ruled out by $((X^1 = 0 \vee X^2 = 0) \wedge (Y^1 = 0 \vee Y^2 = 0))$, i.e. any of the seven transitions into, or out of, the state $(1,1)$. The resulting nine valid configurations lead to a value of $-\log_2(9/16)$ bit.

While all of these partial entropy terms of greater than zero, they cancel out, leaving $i_{\tau sx}^{\{1\}\{2\} \rightarrow \{1\}\{2\}}(\mathbf{X}; \mathbf{Y}) = 0$ bit.
