# Supplementary material for: Decomposing past and future: Integrated information decomposition based on shared probability mass exclusions
Source: PLoS One. 2023 Mar 23;18(3):e0282950. doi: 10.1371/journal.pone.0282950 (PMC10035902; doi:10.1371/journal.pone.0282950)
Supplement: Attachment — Submitted filename: response_to_reviewers.pdf [file pone.0282950.s005.pdf]

We would like to thank the two reviewers for taking the time to evaluate our manuscript and provide critical feedback. Below, our responses are noted in blue font.

Reviewer 1

The article introduces a novel metric of double redundancy and evaluates it in various scenarios. The manuscript is very well written, the proposed metric has a number of desirable properties, and the presented analyses are very interesting. As I believe this is a good contribution to the field, I strongly suggest the manuscript to be accepted for publication.

My main suggestion is the following. It seems to me that this type of information decomposition do not consider negative atoms as a bug, but actually embrace them and provide an explanation for their meaning. If this is the case, I'd like to see that important feature highlighted more through the text. Additionally, it would be nice if this feature could be illustrated a bit more extensively in the analysis of the three synthetic models, where I would have liked to see a bit more of explanation of the meaning of various results.

This is an excellent point, and one where there is a lot of space for discussion and further research. We have added the following:

> "The three systems described above provide concrete toy models which we can use to build intuition about the phenomena of negative ΦI values. Consider the heterogeneous system $\mathbf{S}^H$: specifically the information that S^2_{t-1} communicates to itself at time t ($I(S^2_{t-1}; S^2_t)$). This value, sometimes called active information storage [1], is an expected mutual information and must be non-negative: in this case, it is 0.001 bit. If an observer were just observing the dynamics of S^2 (and ignoring S^1 entirely), their uncertainty about the future of S^2 would be reduced by observing its past. Despite this, the "stored" partial information $\{2\}\{2\}$ is negative. How can this occur?
>
> Our interpretation is that this mismatch is explained by the fact that the evolution of S^2 occurs *in the context of all other elements in the system.* Analysing S^2 on its own can be misleading, because its dynamics are informed by the states of the other elements. In this case, the lions share of the information is information that initially present in both atoms, and then erased from S^1: $\{1\}\{2\} \to \{2\}$. So, a significant amount of the information that one observes in $I(S^2_{t-1}; S^2_t)$ is not *specific* to S^2 (at least not at first), but rather, emerges from the interaction with S^1.
>
> In the case of these toy systems, there is no "mechanism" to be explored, however, this kind of distinction may be of value when analysing real-world systems. For example, a scientist studying the activity of neurons may observe a non-zero active information storage and propose to connect that to biophysical processes such as refractory periods [2], however, the ΦID analysis shows that this information may not actually reflect the specific dynamics of a single neuron, but rather, some contextual interaction between two, and the *actual* memory capacity of the neuron is altogether different (in this case, misinformative, rather than informative). This example is, at present, admittedly speculative, considerable work remains to understand how the outputs of the ΦID algorithm map onto complex, real-world dynamical process."

Our hope is that this comment sheds light on how negative ΦI atoms may be interpreted and reconciled with the non-negativity of Shannon mutual information.

Besides, it seems to me that this Phi-I-D could be readily extended for a local version, i.e. one could use these ideas to define an information decomposition that can be applied to individual datapoints. That could open an exciting line of research studying dynamics with these tools, which perhaps could be mentioned in the discussion? Also, it seems to me that such extension would be more natural than if one tries to do the same with other Phi-I-D decompositions?

The local ΦID analysis formed the lions-share of the avalanche analysis. Section B.2 (titled *Local ΦID Analysis*) describes a frame-wise decomposition over the course of neuronal avalanches.

Finally, I'd suggest to try to introduce the acronym "Phi-I" (which I guess stands for integrated information atom) in the introduction, just to make the reading easier.

This is a good point and we have done so.

Reviewer 2

This paper builds upon the recently introduced Integrated Information Decomposition (phi-ID) from Mediano et al. More specifically, it takes the proposed measure of redundant information I_{sx} from Makkeh et al., which was originally introduced for the Partial Information Decomposition (PID), and proposes a modified measure $I_{\tau sx}$ such that it is now compatible with Phi-ID. The paper discusses some of the properties of the resulting decomposition, and applied it to several examples.

Have reviewed the paper, I have identified 10 significant issues that would need to be addressed before the paper could be accepted. As such, I am recommending that this paper undergo major revisions.

* MAJOR ISSUES / COMMENTS

1. It is not clear to me if Phi-ID is meant purely for the analysis of timeseries, or can it be applied to any random variables (as per PID). Some clarification on this point would be good.

This is an excellent comment - the ΦID is **not** necessarily restricted to temporal dynamics, and could conceivably be applied to any data set with multiple predictor variables and multiple predicted variables (e.g. how might interacting demographic factors disclose information about interacting risk factors). We have expanded part of the Discussion to make this possibility more explicit:

> "...any data set with multiple, interacting predictor and predicted variables: the temporal dimension is not required. This opens up a wider range of applications of data analyses than is accessible to the classic PID - for example, Varley & Kaminski recently used the PID to asses how varying social identities (such as race and sex) jointly disclose information on single outcomes (such as income or health status) [3], however outcomes themselves are not independent and may contain interesting higher-order correlations within themselves. For example, how do the identities race and sex disclose information about income and health outcomes collectively? Generalizing to a ΦID framework may reveal many meaningful dependencies within social data, as well as many other fields where complex systems are studied."

Other, non-temporal applications are also certainly possible.

2. You state that the desire to decompose the excess entropy into separate components associated with the particular elements of the system is the main motivation behind this work, and you describe how such a decomposition could perhaps be used to understand and quantify hitherto weakly defined concepts such as downward and upward causation. This motivation is perfectly reasonable and is of broad interest to the scientific community. You briefly discuss PID before ruling it out because it is only applicable to a single target variable. However, based on what you have written, I don't understand why this is so problematic.

In PID, the target is privileged in the sense we are decomposing the information that the source variables provide about this particular variable. There is no restriction that requires the target variable to be univariate, so in this regard, PID is not limited to a single target variable. Of course, you are well aware of this since (as mentioned at the bottom of page 8) you describe your own work (ref. [18]) which uses PID to decompose the information provided by each state in the past about the entire system in the future. At top of page 9, you state that PID provides no insights into how parts of the system constrain each other, as the future state is aggregated into a single unitary whole. (Actually, you might want to reconsider describing a multivariate variable as a unitary whole.) The problem I have is that clearly PID can also be used to decompose the information provided by each state in the past about any particular state in the future, or indeed about any collection of states in the future. If your application of PID in ref. [18] provides insights into how the states of particular elements collectively constrain the future of the whole system, then why would PID not be able to provide insights into how the states of particular elements constrain particular parts of the system in the future? To me, it seems that you just need to replace the target variable representing the future state of the entire system with a target variable representing the future state of the particular part that you are interested in. Hence, I do not understand how this is supposed to be a fundamental limitation

as you describe it. So, either I misunderstand the problem based on your the description of the problem, in which case you should improve the description, or it is not quite the limitation that you describe.

The reviewer is completely correct in that you could conceivably do the PID of the past state of the whole system onto any particular subset of the system in the future (something like decomposing $I(\mathbf{X}_{-\tau}; X^1_t)$ or $I(\mathbf{X}_{-\tau}; X^2_t)$). We contend that this is a limited approach to decomposing the excess entropy, since it doesn't allow for the possibility of redundant (and by extension, synergistic) interactions among elements in the future. Without some well-defined notion of multi-target redundancy, it is difficult to understand what information disclosed by $I(\mathbf{X}_{-\tau}; X^1_t)$ and $I(\mathbf{X}_{-\tau}; X^2_t)$ is actually common to the future of both of them (i.e. that information that, in the ΦID formulation would belong to the $\{1\} \to \{1\}\{2\}$ or $\{2\} \to \{1\}\{2\}$ atoms). We have added the following:

> "To partially address this issue, one could imagine doing a PID of the information that the joint state of the whole system at time $t - \tau$ discloses every set of elements at time t: decomposing $I(X^1_{-\tau}, \dots X^N_{-\tau}; X^i_t)$, $I(X^1_{-\tau}, \dots X^N_{-\tau}; X^j_t)$, $I(X^1_{-\tau}, \dots X^N_{-\tau}; X^i_t, X^j_t)$, etc. While potentially illuminating, this approach is still limited by the fact that it does not readily allow for notions of "redundancy" and "synergy" between target elements. For example, when computing $I(X^1_{-\tau}, \dots X^N_{-\tau}; X^i_t)$, it might be natural to ask "what information synergistically disclosed by $\mathbf{X}_{-\tau}$ about X^i_t also applies to X^j_t (i.e. is redundantly "copied" over both elements). Similarly, when decomposing $I(X^1_{-\tau}, \dots X^N_{-\tau}; X^i_t, X^j_t)$, one might want to know what information about the joint state of X^i_t and X^j_t could *not* be learned by decomposing $I(X^1_{-\tau}, \dots X^N_{-\tau}; X^i_t)$ or $I(X^1_{-\tau}, \dots X^N_{-\tau}; X^j_t)$ alone (i.e. that information that is synergistically present in the joint state of X^i_t and X^j_t together). To achieve a complete decomposition of the excess entropy, we need to generalize the PID framework to account for redundancies and synergies in both the past *and future* of the system under study."

It is also not at all clear that the result of doing the PID for every combination of elements in the future system could be parsimoniously recombined into the original excess entropy (i.e. it would not provide a true decomposition). For this reason, we think that the ΦID framework is a useful extension that cannot be easily replicated by mix-and-matching PIDs.

3. Following up on the prior point, it seems to me that your problem might perhaps be that decomposing the information provided by each state in the past about a particular state in the future would not strictly speaking be a decomposition of the excess entropy. However, I don't really see how this is a problem. Nevertheless, if it is a problem, then it is worth noting that certain approaches to information decomposition satisfy a chain rule for the target variable. In effect, this means that there is consistency between the decomposition of the information provided by each state in the past about the entire system in the future, and the separate decompositions of the information provided by each state in the past about the particular states in the future. That is to say, if the decomposition satisfying a target chain rule, then the separate decompositions of the information provided by each state in the past about the future state of each particular part in the future, must be consistent with the full decomposition of the full decomposition of the information provided by each state in the past about the future state of the entire system in the future, i.e. the decomposition of the excess entropy. In fact, to me, this consistency seems like a very natural requirement.

To summarise, I don't understand the problem that you say is a limitation of PID, and furthermore, a PID with a target chain rule seems like a much more important problem, as this would me that there is a consistency between the decompositions of the information provided by the past state of the parts about future states of the various parts,and the decomposition of the information provided by the past state of the parts about future state of the entire system. Perhaps you could discuss the relevance of a target chain rule to the problem of decomposition the future states of various parts of the system versus the future state of the entire system.

The possibility of a target chain rule as a solution to the problem of temporal PID is an intriguing one worthy of more extensive study, although I feel it is slightly beyond the scope of this article. We have added

the following brief discussion, however I think this is a deep enough topic to warrant another study of it's own:

> "Another possibility might be to explore temporal PID (rather than ΦID) using a redundancy function equipped with a target chain rule [4]. The target chain rule generalizes the chain rule of mutual information to the PID: a redundancy function $i_\cap$ follows the rule if $i_\cap(\boldsymbol{\alpha}; y_1, y_2) = i_\cap(\boldsymbol{\alpha}; y_1) + i_\cap(\boldsymbol{\alpha}; y_2|y_1)$. Conveniently, the i_{sx} measure satisfies the target chain rule, so a future avenue of research is to compare the results of $i_{\tau sx}$ with the chained i_{sx}."

With that said, I don't think a target chain rule-based decomposition of temporal MI would convey the same information that the Φ ID framework does. My understanding is that conditioning can remove redundancies and reveal synergies, but cannot provide a complete decomposition (see Williams and Beer's discussion about co-information and conditional mutual information). Either way, I feel like this is better left for future work.

4. PID is not yet a well-accepted methodology in information theory. This lack of acceptance is largely down to a lack of a single, well-accepted measure of redundant information that is compatible with the Williams and Beer Axioms. Nevertheless, if one accepts that the Williams and Beer Axioms are reasonable properties for a measure of redundant information, then the derived redundancy lattice cannot be dismissed. To be specific, Williams and Beer start with the Axioms for $I_\cap$, and then show how this axioms reduce the domain of $I_\cap$ to the set of all antichains of the inclusion lattice, i.e. the set A(R) in their notation. They then use the axioms to show that the elements of this set are partially ordered, and that these elements, together with the partial order, form a lattice structure which they call the redundancy lattice. In other words, if you accept the axioms, then you arrive at the redundancy lattice and this is not something that is disputed. Furthermore, Williams and Beer then show how the axioms lead to a two nice properties: specifically, that $I_\cap$ is nonnegative and an increasing function on the redundancy lattice. In this regard, PID is theoretically sound and the fact that we do not yet have a unique measure of redundant information is not problematic until you actually want to evaluate the atoms in the lattice.

Similar to PID, Phi-ID is not generally accepted. However, unlike PID, Phi-ID does not have a simple axiomatic basis. Reference [8] seems to provide two axioms, namely compatibility and partial ordering. It seems to me that the compatibility axiom also contains the original Williams and Beer axioms (although this is not very clearly stated), and so this axiom effectively just states that we have the original Williams and Beer axioms. The second axiom, i.e. partial ordering, however, is not so elegant. In effect, it is a requirement that this double redundancy function satisfies a certain partial ordering, and there isn't much justification provided for this axiom as far as I can tell. Contrast this to PID, where Williams and Beer started with three very simple axioms and then subsequently showed that any function which satisfies these axioms would necessarily be partially ordered. That is to say, in the Williams and Beer approach, the lattice is derived from three simple properties of the redundancy function, whereas in Phi-ID the lattice is effectively introduced as an axiom itself without much justification. In fact, I am actually confused as to how exactly Reference [8] arrives at this lattice structure from their axioms. It seems to come from Proposition 1 in Reference [8], but I do not see any proof of this proposition in their paper.

Strictly speaking, this is a problem with Reference [8] rather than your paper. However, since Reference [8] is not a published paper, this is also problematic for your paper. Since your paper aims to build upon this work, I think it is important that you clarify how exactly the double-redundancy lattice is obtained.

This is a very thoughtful and well-considered critique of the ΦID framework that prompted a considerable amount of consideration on our part. There are a few points we would like to respond to:

The first is that there actually have been a number of published (i.e. peer reviewed) applications of the ΦID in a number of different journals. See:

> Rosas, F. E., Mediano, P. A. M., Jensen, H. J., Seth, A. K., Barrett, A. B., Carhart-Harris, R. L., & Bor, D. (2020). Reconciling emergences: An information-theoretic approach to identify causal emergence in multivariate data. PLOS Computational Biology, 16(12), Article 12. https://doi.org/10.1371/journal.pcbi.1008289
>
> Luppi, A. I., Mediano, P. A. M., Rosas, F. E., Harrison, D. J., Carhart-Harris, R. L., Bor,

D., & Stamatakis, E. A. (2021). What it is like to be a bit: An integrated information decomposition account of emergent mental phenomena. Neuroscience of Consciousness, 2021(2). https://doi.org/10.1093/nc/niab027

Luppi, A. I., Mediano, P. A. M., Rosas, F. E., Holland, N., Fryer, T. D., O'Brien, J. T., Rowe, J. B., Menon, D. K., Bor, D., & Stamatakis, E. A. (2022). A synergistic core for human brain evolution and cognition. Nature Neuroscience, 1–12. https://doi.org/10.1038/s41593-022-01070-0

Mediano, P. A. M., Rosas, F. E., Luppi, A. I., Jensen, H. J., Seth, A. K., Barrett, A. B., Carhart-Harris, R. L., & Bor, D. (2022). Greater than the parts: A review of the information decomposition approach to causal emergence. Philosophical Transactions of the Royal Society A: Mathematical, Physical and Engineering Sciences, 380(2227), 20210246. https://doi.org/10.1098/rsta.2021.0246

And was also presented at the Organization for Computational Neuroscience flagship conference:

`https://cns2020online.sched.com/event/cuJH/w1-s12-multi-target-information-decomposition-and-app`

While I was not privy to the peer-review process of those publications (which span a number of different journals with different scopes, including another PLoS journal), the ΦID framework clearly passed muster for those reviewers. It is not currently quite as unknown as perhaps it was previously.

However, I do think that the points the reviewer made *are* important ones that I have never seem addressed in the literature. We have added the following:

> The ΦID framework deviates from the PID framework in one key way. In the original formulation by Williams and Beer, the lattice (which motivates the Mobius inversion) is derived from the axiomatic properties of the proposed redundancy measure. While there has never been universal agreement on the specific definition of "redundancy", any function that satisfies the original axioms can be shown to induce the antichain lattice. The lattice follows from the definition of redundancy. In contrast, in the ΦID framework, the double-redundancy lattice is not derived from the properties of the $I_{\cap}^{\alpha\to\beta}$ function, but rather, imposed by the decision to take the product of the forward and reveres PI lattices. To address this, Mediano et al., imposed a compatibility on any double-redundancy function [5, 6]. Given two (potentially, but not necessarily) multivariate) variables $\mathbf{X}$, $\mathbf{Y}$ and two double-redundancy atoms $\alpha, \beta \in \mathcal{A}^2$

$$I_{\cap}^{\alpha\to\beta} = \begin{cases} I_{\cap}(\mathbf{X}^{\alpha_1}, \ldots, \mathbf{X}^{\alpha_k}; \mathbf{Y}^{\beta_1}) & \iff |\mathbf{Y}| = 1 \\ I_{\cap}(\mathbf{Y}^{\beta_1}, \ldots, \mathbf{Y}^{\beta_j}; \mathbf{X}^{\alpha_1}) & \iff |\mathbf{X}| = 1 \\ I(\mathbf{X}; \mathbf{Y}) & \iff |\mathbf{X}| = |\mathbf{Y}| = 1 \end{cases}$$

> The compatibility axiom requires that, if one of the variables ($\mathbf{X}$ or $\mathbf{Y}$) is univariate, then the double redundancy function reduces to a classic, single-target redundancy function, and the ΦID reduces to the classic PID.

And later, with respect to the $I_{\tau sx}$ function specifically:

> From the definition of $i_{\tau sx}$ it is clear that it follows the compatibility criteria proposed by Mediano et al., [5, 6]. If we are decomposing $i(\mathbf{x}, \mathbf{y})$ and $|\mathbf{y}| = 1$, then Eq. **??** is equivalent to Eq. **??** as the union of all sources ($\mathbf{b}^1 \cup \ldots \cup \mathbf{b}^m$) is equivalent to the single source y, and likewise for the condition where $|\mathbf{x}| = 1$. This shows that $i_{\tau sx}$ is consistent with both the forward and backwards PIDs (inducing the standard single-target redundancy lattice).

The distinction between the axiomatic approach of Williams and Beer versus the pre-computed product lattice of Mediano and Rosas is a profound one. As it stands, it is beyond the scope of this project (and likely, my ability) to replicate the derivation that Williams did right at this time. Given the apparent success of the ΦID framework in getting published recently, I think it is acceptable to focus on it. However, the reviewer *has* convinced me that ΦID may still be refined and placed on firmer mathematical footing. We leave this for future work, however.

5. Following on from the prior point, you would also need to show that the double-redundancy function that you define satisfies these axioms. If you can't (i) show that the double-redundancy lattice can be derived from the axioms and then (ii) show that your double-redundancy function satisfies these axioms, then you have no justification for later some of the later steps such as the Moebius inversion. You will notice that almost all of the existing proposals for a measure of redundancy for PID first start out by showing that the proposed measure satisfies the Williams and Beer Axioms, or variants thereof.

See our response to Point 4, which covers this issue as well. We have added a discussion of how $I_{\tau sx}$ satisfies the compatibility axiom of Mediano et al., and by extension, satisfies the single-target Williams and Beer axioms (when appropriate).

6. Since you are building so directly upon i_{sx}, I think you need to improved the description of the motivation behind its introduction. As it is, there isn't much beyond the sentence appearing before equation (15). There is a great deal of discussion motivating i_{sx} in the original papers, so it should be expanded since you are basically just extending this approach to make it compatible with Phi-ID.

We have added the following to the introduction of i_{sx}:

> The classic, local mutual information is *bivariate*, quantifying the information shared between two variables. To construct a redundancy function that accounted for multiple sources redundantly disclosing information about a single target, Makkeh et al leveraged a link between redundant information and logical implication [7, 8]. Briefly, given some set of logical statements $\psi^1, \ldots, \psi^k$, the information that is *redundantly* disclosed by all of them is the information learned if ψ^1 is true OR ψ^2 is true OR ... ψ^k is true. From this, they define a logical redundancy measure that induces the same antichain lattice as the PID, with a mapping between every $\alpha \in \mathcal{A}$ and a logical statement. For example, the atom $\{1\}\{2\}\{3\}$ maps to the statement $\psi^1 \vee \psi^2 \vee \psi^3 = True$, and the atom $\{1\}\{2,3\}$ maps to the statement $\psi^1 \vee (\psi^2 \wedge \psi^3) = True$ and so on. The application to random variables is straightforward: given some set of source variables informing on a target y, the information about y redundantly disclosed by all the sources is the information that could be learned by observing x^1 alone OR x^2 alone ... OR x^k alone. The logic extends to more complicated atoms, such as $\{1\}\{2,3\}$, which is the information about y that would be learned by observing just x^1 alone OR the joint state of x^2 AND x^3 together.
>
> As in the case of local mutual information, i_{sx} defines "disclosing information" in terms of probability mass exclusions. For example, observing $X^1 = x^1 \vee (X^2 = x^2 \wedge X^3 = x^3)$ changes the probability of observing $Y = y$. As with the local mutual information, depending on how $P(y)$ changes, the value of i_{sx} can be positive or negative.

7. Similarly, when you introduce $I_{\tau sx}$, you do not provide much of a description of the motivation behind this particular definition. In particular, you should be justifying the replacement that you make in your definition relative to the original definition of i_{sx} provided in equation (15). Furthermore, is this change obvious? I am not saying that it is, but since the original i_{sx} was proposed for PID rather than Phi-ID, then i_{sx} would clearly also need to be modified. Perhaps the change you have made it a trivial change, or maybe it is more significant in some way. Either case is fine, but you really aught to be discussing this change and justifying why you have defined this generalisation in this particular way.

We have added the following:

> It is worth considering the intuition behind this change. Suppose $\mathbf{x} = \{x^1, x^2\}$ and $\mathbf{y} = \{y^1, y^2\}$. We are interested in the double redundancy $i_{\tau sx}(x^1, x^2; y^1, y^2)$: the information about y^1 OR y^2 that could be learned by observing x^1 OR x^2. There are some global configurations of $\{\mathbf{x},\mathbf{y}\}$ that are be consistent with either y^1 OR y^2 (given by $P(y^1 \cup y^2)$). If we exclude all of those past-future configurations that are also *not* consistent with x^1 OR x^2 (i.e. *are* consistent with $\bar{x}^1 \cap \bar{x}^2$), the double-redundancy is a function of the relative change in $P(y^1 \cup y^2)$. Formally:

As for whether this is a trivial or significant, I don't really feel like I can rule on that one way or another. It's certainly not a tremendous change to the function, but it also (in my opinion) opens a lot of doors that "vanilla" i_{sx} could not.

8. You are proposing a decomposition that is based on the local mutual information, which is a non-negative function. The justification for doing this seems to come from reference [35] which gets around this issue by decomposing the local entropy instead of the local mutual information, i.e. the local mutual information is first decomposed into two non-negative local entropies, and then each of these local entropies are decomposed separately. Thus, really you have two separate information decompositions (i.e. two separate decompositions of the local entropy), and you are combining the corresponding atoms of these two separate decompositions quantities to get local-mutual-information-like quantities. In other words, you are not really decomposition the local mutual information, but rather you are decomposing the two local entropies.

You present your decomposition as though it is decomposition of the local mutual information, and make it sound like the decomposition of the local entropies is an additional feature. To me, it seems like it is actually a decomposition of the local entropy, and you are also combining corresponding atoms of these two separate decompositions quantities to get local-mutual-information-like quantities. This difference is important as the results from the recombine atoms really should be seen as the results obtained from combining quantities from two separate decompositions, rather than a decomposition in its own right. This problem is also present in reference [10].

In this regard, I was following the pattern originally set by Makkeh et al., when they introduced the I_{sx} measure, which in turn followed from the work by Connor Finn in the work published from his PhD thesis. There, he established the convention of referring to the entropic components of local mutual information as i^+ and i^- respectively. For consistency with prior literature, I would like to continue with this pattern.

We have revisited the notion of the partial entropy decomposition, and re-written a large section, adding the following:

> The full $i_{\tau sx}^{\boldsymbol{\alpha}\to\boldsymbol{\beta}}$ function does not satisfy the partial ordering (monotonicity) criteria, however. Like i_{sx}, it can be negative or positive, depending on the structure of the dependency between the elements. The double-redundancy *can* be decomposed, however, into three *redundant entropy terms* that are non-negative, partially ordered, and consistent with the integrated information lattice. These three redundant entropy terms induce three partial entropy decompositions [9, 10]: two marginal decompositions on the classic redundancy lattice, and a joint decomposition on the product lattice.

0.0.1 Multi-Target Redundancy & Entropy Decomposition

The double redundancy function $i_{\tau sx}^{\boldsymbol{\alpha}\to\boldsymbol{\beta}}$ can be re-written in terms of sums and differences of union entropies. We can re-write Eq. **??** in an equivalent form:

$$
\begin{aligned}
i_{\tau sx}(\boldsymbol{\alpha}\to\boldsymbol{\beta}) = \\
&\log_2 \frac{1}{P(\mathbf{a}^1 \cup \ldots \cup \mathbf{a}^k)} \\
&+\log_2 \frac{1}{P(\mathbf{b}^1 \cup \ldots \cup \mathbf{b}^m)} \\
&-\log_2 \frac{1}{P((\mathbf{a}^1 \cup \ldots \cup \mathbf{a}^k) \cap (\mathbf{b}^1 \cup \ldots \cup \mathbf{b}^m))}
\end{aligned} \tag{1}
$$

For proof of equivalence, see Appendix A.

For some multivariate $\mathbf{x}$, recall that $i(\mathbf{x};\mathbf{x}) = h(\mathbf{x})$. We can decompose the joint entropy $h(\mathbf{x})$ by assessing how different combinations of *parts* (i.e. all $x^i \in \mathbf{x}$) redundantly and synergistically disclose information about the *whole* [8]. If we use the single-target i_{sx} function to decompose

$i(x^1, \ldots, x^k; \mathbf{x})$, we will find that it is equal to $i^+(x^1, \ldots, x^k; \mathbf{x})$: there is no misinformative component (for proof, see Appendix B).

Intuitively, we can understand the redundant entropy function as quantifying how much uncertainty about $\mathbf{x}$ is resolved by learning $x^1 \vee \ldots \vee x^k$. We will denote the this as the redundant entropy function h_{sx}, after [8]:

$$h_{sx}(\boldsymbol{\alpha}) = \log_2 \frac{1}{P(\mathbf{a}^1 \cup \ldots \cup \mathbf{a}^k)} \tag{2}$$

Which has been previously shown to satisfy the relevant Williams and Beer axioms locally [8]. We can then re-write Eq. **??** as:

$$i_{\tau sx}(\boldsymbol{\alpha} \rightarrow \boldsymbol{\beta}) = h_{sx}(\boldsymbol{\alpha}) + h_{sx}(\boldsymbol{\beta}) - h_{sx}(\boldsymbol{\alpha} \cap \boldsymbol{\beta}) \tag{3}$$

This framing provides a different, but complementary perspective on $i_{\tau sx}$. The first term, $h_{sx}(\boldsymbol{\alpha})$ quantifies how uncertainty about the global joint state ($\mathbf{x}$,$\mathbf{y}$) is resolved by learning past states $\mathbf{a}^1$ OR $\ldots \mathbf{a}^k$ etc. Similarly, $h_{sx}(\boldsymbol{\beta})$ quantifies the uncertainty about ($\mathbf{x}$,$\mathbf{y}$) resolved by learning future states $\mathbf{b}^1$ OR$\ldots \mathbf{b}^m$.

The final term, $h_{sx}(\boldsymbol{\alpha} \cap \boldsymbol{\beta})$ is a little bit less straightforward, and reflects the structure of the double redundancy lattice. We can show that it satisfies the required partial ordering imposed by Mediano et al., [5]. From [8], we know that, if $\boldsymbol{\alpha} \preceq \boldsymbol{\alpha}'$ on the marginal (classic) redundancy lattice, then the set of configurations consistent with $\boldsymbol{\alpha}'$ is a subset of those configurations consistent with $\boldsymbol{\alpha}$. This ensures that $h_{sx}(\boldsymbol{\alpha}) \leq h_{sx}(\boldsymbol{\alpha}')$, and likewise for $\boldsymbol{\beta}$. The double redundant entropy term $h_{sx}(\boldsymbol{\alpha} \cap \boldsymbol{\beta})$ quantifies the probability of the *intersection* of the configurations consistent with $\boldsymbol{\alpha}$ AND $\boldsymbol{\beta}$. If $\boldsymbol{\alpha}' \subseteq \boldsymbol{\alpha}$ and $\boldsymbol{\beta}' \subseteq \boldsymbol{\beta}$, then $\boldsymbol{\alpha}' \cap \boldsymbol{\beta}' \subseteq \boldsymbol{\alpha} \cap \boldsymbol{\beta}$ and consequently, $h_{sx}(\boldsymbol{\alpha} \cap \boldsymbol{\beta}) \leq h_{sx}(\boldsymbol{\alpha}' \cap \boldsymbol{\beta}')$. For a worked example, see Appendix C.

From Eq. 3 we can also construct the informative and misinformative probability mass exclusion formulation of $i_{\tau sx}$ equivalent to the informative and misinformative components of i_{sx}, however unlike in the single-target case, $i^-_{\tau sx}$ does not follow the partial ordering criteria that h_{sx} does and is also not strictly non-negative.

9. The synthetic systems that you use here in this paper: where do they come from? Are you introducing them for the first time here, or are they well known within the field of IIT? Do we know what the actual results should be or are their certain results which are seen as desirable based on intuition?

The systems were "designed" by me to explore the limit cases of Φ^{WMS}'s behavior: one where the whole is trivially reducible to the parts (the disintegrated system), one where the whole was entirely irreducible to the parts (the integrated system), and one that showed a mix of the two. These are, to the best of my knowledge, not canonical systems, although they're simple enough that any readers familiar with PID or IIT should get the gist of them. We have added the following:

> "These systems were designed to show the two extreme behaviours of Φ^{WMS}: the first system is totally dis-integrated and $\Phi^{WMS} = 0$ bit. The second system is completely integrated: the sum of the excess entropy of the parts is 0 bit, while the whole is has non-zero excess entropy. The third system is a heterogeneous combination of integrated and dis-integrated dynamics. Considering the limiting cases of Φ^{WMS}, we can build intuition about how the ΦID framework with $I_{\tau sx}$ describes diverse kinds of systems. We hypothesized that the disintegrated system should, generally, not have much redundant temporal mutual information, as the elements are independent of each-other, and there should be little information transfer between individual elements. Similarly, in the case of the integrated system, we expected low redundancy, and a high degree of synergy (as the future of the whole can only be partially predicted by knowing the past of the whole)."

In general, going into the toy models, we did **not** know exactly what the results should be (although they could be done by hand). This is part of what makes them an interesting starting point, however: they can demonstrate where the ΦID framework diverges from our intuitions, and are simple enough that those divergences might be explainable.

In general, your description of obtain results does not give me the impression that the results are well understood. For instance, when it comes to describing the result for the disintegrated system, you describe the the element-wise storage atoms as being the "strongest" while the pairwise transfer atoms are negative. You describe this latter result as being "consistent with the notion of the system as a disintegrated structure", but I don't see how this is the case and you do not provide any further clarification. You say that there are more positive interactions(?) between individual elements and higher-order synergistic joint states than anticipated. However, you never described or justified your anticipated result, so this is not a helpful comment to make. The fact that you describe this result a "curious" does not fill me with confidence that the results are indeed sensible or helpful. These results are all local mutual informations, but as described in point 7, these values are really obtained from separate decompositions of the two local entropies, so I suspect these results would be less mysterious if you looked at these values.

Similarly, you are also a bit vague and uncertain about the results obtained from the integrated system. Do we know what the results should be, or are is there some kind of intuitive result that you would like to see? Describing some of the obtained results as a "surprise" when you are considering synthetic examples that should have some definite result is quite problematic to me. This could be fine if you provide an explanation for the surprising results, but I don't see any justification that explains these results to me. This kind of description would be absolutely fine if this was a well established method and you were applying it to some empirical data from some system that is not well understood. It is troubling, however, when these are the results that you obtain from applying this to synthetic examples which really should be justifying the theoretical definition that you have introduced.

Regarding the heterogeneous system, I am not really sure what we are supposed to take away from this example. You were actually a bit more explicit in stating your expectation for this example, in that you said that you expected to see multiple types of information atoms present, and indeed, this is the result that you obtained. However, what does it really tell us to learn that a randomly generated example produces a dynamic which is associated with a small amount of information associated with each of the atoms? Without further guidance, I am not sure what exactly I should take away from this.

There are a few points to respond to here. The first is that I had initially tried to err on the side of caution when it came to interpreting the results (which perhaps came off as waffling). My concern was "over interpreting" what I saw. However, I have re-written the relevant sections to more emphatically reflect my interpretations.

> Each of the three synthetic system is comprised of two, binary, elements that evolve through times according to different Markovian state-transition networks (visualized in Fig. **??**). Prior work on such simple, Boolean networks has shown that the space of even very small systems has a surprisingly rich distributions of redundant, unique, and synergistic effective information atoms [11]. Despite the simplicity of the synthetic systems under study here, we can see how $I_{\tau sx}$ can reveal markedly different dynamic regimes. These systems were designed to show the two extreme behaviours of Φ^{WMS}: the first system is totally dis-integrated and $\Phi^{WMS} = 0$ bit (as the future of the whole can be perfectly predicted from the independent parts). The second system is completely integrated: the sum of the excess entropy of the parts is 0 bit, while the whole is has non-zero excess entropy. The third system is a heterogeneous combination of integrated and dis-integrated dynamics. Considering the limiting cases of Φ^{WMS}, we can build intuition about how the ΦID framework with $I_{\tau sx}$ describes diverse kinds of systems. We hypothesized that the disintegrated system should, generally, not have much redundant temporal mutual information, as the elements are independent of each-other, and there should be little information transfer between individual elements. Similarly, in the case of the integrated system, we expected low redundancy, and a high degree of synergy (as the future of the whole can only be partially predicted by knowing the past of the whole).

0.0.2 Disintegrated System

The first system, $\mathbf{S}^D$ is a "disintegrated" system, in that each of the two dynamic elements is disconnected from the other: both predict *their own* futures with total determinism (the pattern is an oscillation $1 \to 0 \to 1 \to \ldots$), however there is no integration. Consequently, the excess entropy $I(\mathbf{S}^D_{t-1};\mathbf{S}^D_t) = 2$ bit, and both individual excess entropies are each 1 bit: the "whole" is trivially reducible to the sum of its parts, since there's no actual interaction between elements. For a visualization of the state-transition matrix, see Figure **??**, left.

Decomposing the excess entropy using $I_{\tau sx}$ reveals several interesting relationships (for the full decomposition, see Table **??**). As expected, the strongest information atoms are the element-wise information "storage" atoms: $\{1\} \to \{1\}$ and $\{2\} \to \{2\}$ atoms. This is consistent with our intuition that the future of each element is *best* predicted by its own immediate past. The fact that the unique information storage atoms are also the largest single atoms is consistent with the idea that the most informative information dynamic about the whole system is the behaviour of the individual nodes considered individually. The informative interaction between the unique information and the synergistic information are also consistent with our intuitions about the disintegrated systems. For instance, the informative value of the "upward-causation" atom $\{1\} \to \{12\}$ reflects the fact that knowing the state of $X^1_{-\tau}$ also informs on the state of the whole $\mathbf{X}_t$ in the future: knowing $X^1_{-\tau} = 0$ rules out any configuration of $\mathbf{X}_t$ where $X^t = 0$ (since the values of each X^i oscillate). Likewise for the "downward-causation" atoms such as $\{12\} \to \{1\}$: knowing the state of the *whole* at time $t - \tau$ constrains the individual parts at time t (albeit, to a lesser extent than they constrain themselves).

The non-zero value of the double redundancy atom $\{1\}\{2\} \to \{1\}\{2\}$ is unexpected, although not inexplicable. Suppose that, at time $t - \tau$ we learn that $X^1_{-\tau} = 0$ OR $X^2_{-\tau} = 0$. Since both X^i oscillate $0 \to 1 \to 0 \to 1 \ldots$, learning the state of either past variable is enough to rule out one possible joint future: $\mathbf{X}_t = (0, 0)$. Consequently, the union probability of all possible futures consistent with $X^1_t \neq 0$ OR $X^2_t \neq 0$ increases.

The final set of atoms worth exploring are the negative value for the information transfer terms (such as $\{1\} \to \{2\}$). The temporal mutual information $I(X^1_{-\tau};X^2_t) = 0$ bit, but the partial information atom is less than zero. Why? The answer is that computing the information transfer atom requires subtracting the sum of all the atoms that precede it on the lattice from the temporal mutual information. In this case, this is only the non-zero, double redundancy atom $\{1\}\{2\} \to \{1\}\{2\}$, which when subtracted off, produces a negative value (since the temporal mutual information is 0 bit).

0.0.3 Integrated System

The second system, $\mathbf{S}^G$ is an "integrated" system, in that there the whole system has 1 bit of excess entropy, but both elements have individual excesses entropies of 0 bit. This is accomplished using a parity check function: at every time step, the parity of the system is preserved, but the individual assignments are done randomly. For example, if $\mathbf{S}^G_{-\tau} = (1, 0)$, then $\mathbf{S}^G_t$ could equal $(0, 1)$ or $(1, 0)$ with equal probability (but never $(0, 0)$ or $(1, 1)$). For a visualization of the state-transition matrix, see Figure **??**, center.

The first interesting finding is that, as with $\mathbf{S}^D$, the double-redundancy atom is unexpectedly positive. This occurs because, as before, learning $X^1_{-\tau}$ OR $X^2_{-\tau}$ is sufficient to inform on the future. Suppose we learn that $X^1_{-\tau} = 0$ OR $X^2_{-\tau} = 0$. There are two configurations with an odd-parity consistent with those conditions ((0,1) and (1,0)), and only one configuration with even parity ((0,0)). From this, we are lead to believe that the future state will also be more likely to have an odd parity than an even one. Due to the synergistic nature of the parity-check function, we cannot directly project from the parity of the joint state back down onto the states of the individual elements, which explains the negative values of the various information copy and erase atoms (such as $\{1\} \to \{1\}\{2\}$ and $\{1\}\{2\} \to \{1\}$). The non-negative values of the various unique information storage and transfer atoms ($\{1\} \to \{1\}$ and $\{1\} \to \{2\}$) occurs

because the mutual informations between any pair of elements are all 0 bit (i.e. $I(X^1_{-\tau}; X^1_t) =$ 0 bit, $I(X^1_{-\tau}; X^2_t) =$ 0 bit, etc), however, the sum of all atoms lower down on the lattice is negative. For example, consider atom $\{1\} \to \{1\}$. The excess entropy $E(X^1) = 0$ bit, however $\{1\}\{2\} \to \{1\}\{2\} + \{1\}\{2\} \to \{1\} + \{1\} \to \{1\}\{2\} = -0.152$ bit, so the value of $\{1\} \to \{1\}$ works out to $0 - (-0.152)$ bit: 0.152. The result can be admittedly difficult to interpret, although negative atoms in classic, single-target PIDs are also relatively widespread (see [12, 13, 8]). One possible interpretation, explored more in Section 0.0.5 is that these values reflect how the *context* provided by the dynamics of the whole system can influence our interpretation of dynamics of the individual parts. For example, the when considered alone, there is no predictive information about the future of X^1 in it's own past, however, X^1's evolution through time is not an autonomous process, but occurs *in the context of* X^2's dynamics. Consequently, the kinds of inferences we can make about X^1 when we consider the other elements of the system may be different from the kinds of inferences we would make if we were considering X^1 alone.

The higher-order, synergistic atoms show the same effect: since the redundancy-to-synergy atoms ($\{1\}\{2\} \to \{12\}$ and $\{12\} \to \{1\}\{2\}$) are both negative, the upward and downward-causation atoms are positive, despite the fact that the relevant mutual informations are all zero bit.

0.0.4 Heterogeneous System

The final system was one with heterogeneous transitions, with probabilities drawn from a Gaussian distribution $\mathcal{N}(0, 1)$ (for details, see Varley & Hoel [11]). In contrast to the prior two systems, this system, $\mathbf{S}^H$ does not have an *a priori* fixed "type" of dynamic and was expected to display multiple types of information conversion. From the outset, we anticipated evidence of synergistic dynamics, as the excess entropy of the whole system was 0.422 bit, while each of the two elements had individual temporal mutual informations of 0.017 bit and 0.001 bit respectively, indicating a dynamic where the whole is much more predictive than the sum of its parts. For a visualization of the state-transition matrix, see Figure **??**, right.

Consistent with expectations, $\mathbf{S}^H$ did not have the same regularity of information dynamics displayed by $\mathbf{S}^D$ and $\mathbf{S}^G$: for example, the atom $\{1\}\{2\} \to \{1\}$ was negative, indicating a misinformative relationship, while $\{1\}\{2\} \to \{2\}$ was positive and had a greater absolute value. Similarly, the conversion from redundant to synergistic information and vice versa both has opposite signs, suggesting that this system simultaneously displays informative "downward causation", but misinformative "upward causation". In totality, there were more informative integrated information atoms than misinformative ones (a ratio of 11 to 5), showing that, despite the overall strongly synergistic nature of the system, unique information transfer and redundant information dynamics all co-existed together. This is consistent with previous work that found that these kinds of modified-Gaussian systems can display a wide range of information dynamics, at multiple scales [11].

0.0.5 Interpreting Negative Local ΦI Atoms

The three systems described above provide concrete toy models which we can use to build intuition about the phenomena of negative ΦI values. Consider the heterogeneous system $\mathbf{S}^H$: specifically the information that S^2_{t-1} communicates to itself at time t ($I(S^2_{t-1}; S^2_t)$). This value, sometimes called active information storage [1], is an expected mutual information and must be non-negative: in this case, it is 0.001 bit. If an observer were just observing the dynamics of S^2 (and ignoring S^1 entirely), their uncertainty about the future of S^2 would be reduced by observing its past. Despite this, the "stored" partial information $\{2\}\{2\}$ is negative. How can this occur?

Our interpretation is that this mismatch is explained by the fact that the evolution of S^2 occurs *in the context of all other elements in the system.* Analysing S^2 on its own can be misleading, because its dynamics are informed by the states of the other elements. In this case, the lions share of the information is information that initially present in both atoms, and then erased from

S^1: $\{1\}\{2\} \rightarrow \{2\}$. So, a significant amount of the information that one observes in $I(S^2_{t-1}; S^2_t)$ is not *specific* to S^2 (at least not at first), but rather, emerges from the interaction with S^1.

In the case of these toy systems, there is no "mechanism" to be explored, however, this kind of distinction may be of value when analysing real-world systems. For example, a scientist studying the activity of neurons may observe a non-zero active information storage and propose to connect that to biophysical processes such as refractory periods [2], however, the ΦID analysis shows that this information may not actually reflect the specific dynamics of a single neuron, but rather, some contextual interaction between two, and the *actual* memory capacity of the neuron is altogether different (in this case, misinformative, rather than informative). This example is, at present, admittedly speculative, considerable work remains to understand how the outputs of the ΦID algorithm map onto complex, real-world dynamical process.

10. The empirical example using a dissociated neural culture: I don't want to be overly negative here because I think the analysis of this kind of data is an interesting scientific endeavour, and I think that information-theoretic methodologies are an appropriate way of potentially understanding this kind of system. However, this entire section to me, is not helpful for a paper that aims to propose a novel theoretical approach. It is interesting, but I think that this work would be far more appropriate as a separate paper after this theoretical method has been accepted in its own right.

The theoretical result should be interesting based on its own merits. You should propose the theoretical measure, provide the motivation for the definition, and justify it using well-understood examples. Typically, for this kind of proposal, you would typically use synthetic examples (indeed, as you have aimed to do with your synthetic examples). This is certainly true for all of the PID based approaches, although perhaps it is more tricky for Phi-ID (see point 1).

The problem here is that the motivation for defining your measure is not very clear (see points 6 and 7), and I think there are some notable theoretical shortcomings (see points 4, 5 and 8). Then the results the you obtain for the synthetic examples are not clear or convincing (see point 9). With all of this in mind, I do not see what value the empirical example where there is no well-established ground truth. If the results can't be fully explained for the synthetic examples that are designed to exhibit certain features or characteristics, then I don't see how we can trust the results when applied to real empirical data.

On the flip side, it is you paper, so I don't want to say that this analysis must be removed. Rather, I am saying that it needs much better theoretical justification before we can trust the results, or draw inference from the results when applied to empirical data. To reiterate, the theoretical results should be interesting in there own right, and should stand on their own two feet (to use an idiom). In my opinion, the level of detail required achieve this level of theoretical rigour would result in a paper in its own right, and then this example could potentially go in a separate empirical paper that uses the newly establish theoretical framework. Whether or not you want to keep the empirical example in this paper, you must improve the rigour of the theoretical results before this paper is suitable for publication.

I feel quite strongly that the empirical results should be kept. I think it is important that there be at least *some* application to a real-world system to show that this is not simply a theoretical exercise. This was inspired by the original introduction of ΦID by Mediano et al., who worked through the derivation of the double-redundancy lattice, and then ended with a small number of "real-world" applications, including an analysis of invasive ECoG recordings from macaques.

There, as in here, the goal was not to claim a ground-breaking new result, but rather, to provide a kind of proof-of-concept that could inspire interested researchers and demonstrates how the analysis could be applied. The best of our knowledge, this kind of frame-wise, local information-theoretic analysis of avalanche profiles has never been done in neural cultures. The hope is that the work presented here will motivate future work that *can* dig into the gory detail more. In this context, the fact that the results are largely descriptive is not necessarily a deal-breaker.

References

[1] Joseph T. Lizier. *The Local Information Dynamics of Distributed Computation in Complex Systems.* Springer Theses. Springer Berlin Heidelberg, Berlin, Heidelberg, 2013.

[2] Thomas F. Varley, Olaf Sporns, Stefan Schaffelhofer, Hansjörg Scherberger, and Benjamin Dann. Information processing dynamics in neural networks of macaque cerebral cortex reflect cognitive state and behavior, May 2022. Section: New Results Type: article.

[3] Thomas F. Varley and Patrick Kaminski. Untangling Synergistic Effects of Intersecting Social Identities with Partial Information Decomposition. *Entropy*, 24(10):1387, October 2022. Number: 10 Publisher: Multidisciplinary Digital Publishing Institute.

[4] Nils Bertschinger, Johannes Rauh, Eckehard Olbrich, and Jürgen Jost. Shared Information – New Insights and Problems in Decomposing Information in Complex Systems. *arXiv:1210.5902 [cs, math]*, pages 251–269, 2013. arXiv: 1210.5902.

[5] Pedro A. M. Mediano, Fernando Rosas, Robin L. Carhart-Harris, Anil K. Seth, and Adam B. Barrett. Beyond integrated information: A taxonomy of information dynamics phenomena. *arXiv:1909.02297 [physics, q-bio]*, September 2019. arXiv: 1909.02297.

[6] Pedro A. M. Mediano, Fernando E. Rosas, Andrea I. Luppi, Robin L. Carhart-Harris, Daniel Bor, Anil K. Seth, and Adam B. Barrett. Towards an extended taxonomy of information dynamics via Integrated Information Decomposition. *arXiv:2109.13186 [physics, q-bio]*, September 2021. arXiv: 2109.13186.

[7] A. J. Gutknecht, M. Wibral, and A. Makkeh. Bits and pieces: understanding information decomposition from part-whole relationships and formal logic. *Proceedings of the Royal Society A: Mathematical, Physical and Engineering Sciences*, 477(2251):20210110, July 2021. Publisher: Royal Society.

[8] Abdullah Makkeh, Aaron J. Gutknecht, and Michael Wibral. Introducing a differentiable measure of pointwise shared information. *Physical Review E*, 103(3):032149, March 2021. Publisher: American Physical Society.

[9] Robin A. A. Ince. The Partial Entropy Decomposition: Decomposing multivariate entropy and mutual information via pointwise common surprisal. *arXiv:1702.01591 [cs, math, q-bio, stat]*, February 2017. arXiv: 1702.01591.

[10] Conor Finn and Joseph T. Lizier. Generalised Measures of Multivariate Information Content. *Entropy*, 22(2):216, February 2020. Number: 2 Publisher: Multidisciplinary Digital Publishing Institute.

[11] Thomas F. Varley and Erik Hoel. Emergence as the conversion of information: a unifying theory. *Philosophical Transactions of the Royal Society A: Mathematical, Physical and Engineering Sciences*, 380(2227):20210150, July 2022. Publisher: Royal Society.

[12] Robin A. A. Ince. Measuring Multivariate Redundant Information with Pointwise Common Change in Surprisal. *Entropy*, 19(7):318, July 2017. Number: 7 Publisher: Multidisciplinary Digital Publishing Institute.

[13] Conor Finn and Joseph T. Lizier. Pointwise Partial Information Decomposition Using the Specificity and Ambiguity Lattices. *Entropy*, 20(4):297, April 2018. Number: 4 Publisher: Multidisciplinary Digital Publishing Institute.
