# Supplementary material for: Decomposing past and future: Integrated information decomposition based on shared probability mass exclusions
Source: PLoS One. 2023 Mar 23;18(3):e0282950. doi: 10.1371/journal.pone.0282950 (PMC10035902; doi:10.1371/journal.pone.0282950)
Supplement: Attachment — Submitted filename: Refere Report.pdf [file pone.0282950.s006.pdf]

Report on:

Decomposing past and future: Integrated information decomposition based on shared probability mass exclusions.

In this paper new information-theoretic metric is described which quantifies the how much redundant information is shared between sources and targets. It is built upon recently introduced Integrated Information Decomposition for multiple targets, which is a generalization of Partial Information Decomposition. Section 1 carefully follows the previous developments of Integrate and Partial Information Decomposition. This sets the stage for section 2 where which describes the measure of single-target redundancy and finally multi-target redundancy based on shared exclusions ($I_{\tau sx}$). In section 3, $I_{\tau sx}$ is used to decompose the excess entropy in synthetic data as well as empirical data from dissociated neural culture. Finally, there is a discussion of $I_{\tau sx}$ and also its limitations.

This article is well written and does a good job building up all the necessary pieces to define $I_{\tau sx}$. The applications demonstrate its utility and there is a thoughtful discussion afterwards.

The authors were able to add more to clarify questions from other reviews, and I believe each comment has been addressed. I have no further questions or notes for the work and am recommending it to be accepted.
